# Protein Phosphatase 2A Promotes CD8^+^ T Cell Effector Function through the Augmentation of CD28 Costimulation

**DOI:** 10.34133/research.0545

**Published:** 2025-01-02

**Authors:** Kaixiang Zhu, Deepak Rohila, Yuanling Zhao, Dmytro Shytikov, Lize Wu, Fan Zhao, Shurong Hu, Qin Xu, Xuexiao Jin, Linrong Lu

**Affiliations:** ^1^ Department of Cardiology of The Second Affiliated Hospital, Zhejiang University School of Medicine, Hangzhou 310009, China.; ^2^ State Key Laboratory of Transvascular Implantation Devices, Hangzhou 310009, China.; ^3^ Heart Regeneration and Repair Key Laboratory of Zhejiang Province, Hangzhou 310009, China.; ^4^ Institute of Immunology, and Department of Rheumatology in Sir Run Run Shaw Hospital, Zhejiang University School of Medicine, Hangzhou 310058, China.; ^5^ Sanford Burnham Prebys Medical Discovery Institute, San Diego, CA, USA.; ^6^ Zhejiang University–University of Edinburgh Institute, Zhejiang University School of Medicine, 314400 Haining, China.; ^7^ Department of Gastroenterology, The Second Affiliated Hospital, Zhejiang University School of Medicine, Hangzhou, China.; ^8^ Cell Signaling and Immunity Section, Laboratory of Immune System Biology (LISB), National Institute of Allergy and Infectious Diseases (NIAID), National Institutes of Health (NIH), Bethesda, MD, USA.; ^9^ Shanghai Immune Therapy Institute, Shanghai Jiao Tong University School of Medicine Affiliated Renji Hospital, Shanghai 200025, China.

## Abstract

Protein phosphatase 2A (PP2A) is one of the most abundant serine/threonine phosphatases and plays critical roles in regulating cell fate and function. We previously showed that PP2A regulates the differentiation of CD4^+^ T cells and the development of thymocytes. Nevertheless, its role in CD8^+^ T cells remains elusive. By ablating the catalytic subunit α (Cα) of PP2A in CD8^+^ T cells, we revealed the essential role of PP2A in promoting the effector functions of CD8^+^ T cells. Notably, PP2A Cα-deficient CD8^+^ T cells exhibit reduced proliferation and decreased cytokine production upon stimulation in vitro. In vivo, mice lacking PP2A Cα in T cells displayed defective immune responses against lymphocytic choriomeningitis virus infection, associated with reduced CD8^+^ T cell expansion and decreased cytokine production. Consistently, the ablation of the PP2A Cα subunit in CD8^+^ T cells results in attenuated antitumor activity in mice. There is a notable decrease in the infiltration of PP2A Cα-deficient CD8^+^ T cells within the tumor microenvironment, and the cells that do infiltrate exhibit diminished effector functions. Mechanistically, PP2A Cα deficiency impedes CD28-induced AKT Ser^473^ phosphorylation, thus impairing CD8^+^ T cell costimulation signal. Collectively, our findings underscore the critical role of phosphatase PP2A as a propeller for CD28-mediated costimulation signaling in CD8^+^ T cell effector function by fine-tuning T cell activation.

## Introduction

CD8^+^ T cells play a crucial role in defending the body against infections and cancer by responding to various activation signals, such as the T cell receptor (TCR) and co-receptor (CD28) [1–4]. Signal transduction encompasses the process of transmitting and converting signals during T cell activation, which dictates the function and differentiation of CD8^+^ T cells [2]. Phosphatases are key enzymes that modulate the activity and function of proteins by regulating the phosphorylation status of proteins, thereby affecting both intracellular and extracellular signaling processes [5,6].

The major Ser/Thr phosphatase protein phosphatase 2A (PP2A) controls multiple cellular processes including cell proliferation, survival, differentiation, and function. PP2A is responsible for a minimum of half of all Ser/Thr dephosphorylation in the majority of cell types, existing mainly as trimeric holoenzymes made up of catalytic (C), scaffolding (A), and variable regulatory (B) subunits [7]. PP2A can form nearly 100 distinct subunit combinations in mammalian cells, thereby determining specific localizations, substrates, and regulatory mechanisms [8]. The PP2A Cα isoform of PP2A catalytic subunit (PP2Ac) is the dominant catalytic subunit, which plays a functional role in over 90% of PP2A complexes [9]. Due to its abundant expression and various role in cellular process, previous studies have tried to discuss the precise role of PP2A in T cell activation. PP2A was initially characterized as a negative regulator of T cell activation, modulating transmembrane signaling triggered by TCR engagement [9,10]. The antigen-specific cytotoxic potential of lymphocytes was marked amplified following PP2A inhibition [10]. A study also demonstrated that inhibiting the regulatory subunit of PP2A (PPP2R2D) using short hairpin RNA (shRNA) increases the growth, cytokine release, and killing abilities of effective CD4^+^ and CD8^+^ T cells, as well as tumor-infiltrating lymphocytes (TILs) when these cells are transferred into the recipient mice. This augmentation thereby boosts the antitumor effect in the B16-OVA melanoma mouse model [11,12]. In addition, hypermethylation of PPP2R2B leads to acquired apoptosis deficiency in systemic autoimmune diseases [13]. Another study also showed that in activated T cells, PP2A could cause dephosphorylation and then inactivation of AKT by mediating the CTLA-4 inhibitory signaling pathway [14].

Our group’s focus lies in investigating the role of PP2A Cα within the T cell lineage. Previously, we confirmed that PP2A Cα plays a crucial role in thymocyte development by regulating cell survival [15]. PP2A Cα has been shown to increase the pro-inflammatory capacity of CD4^+^ T cells by facilitating interleukin-17 (IL-17) production [16,17]. Furthermore, we demonstrated that differentiation of T helper 17 (T_H_17) cells was significantly impaired when PP2A was deleted from mature T cells, indicating that blocking PP2A Cα may be a viable treatment approach for controlling T_H_17 cell-associated autoimmune diseases [18]. We further elucidated that PP2A Cα affects T follicular helper (T_FH_) cell differentiation and underscored the therapeutic potential of targeting PP2A Cα in the treatment of systemic lupus erythematosus (SLE) [19]. PP2A has been shown to be crucial for the proper functioning of regulatory T cells and for preventing autoimmune reactions, as reported in a study [20]. In addition to its pronounced expression in CD4^+^ T cells, PP2A also exhibits notable expression levels within CD8^+^ T cell populations. PP2A B55β has been demonstrated to play a pivotal role in IL-2 withdrawal-induced apoptosis in CD8^+^ T cells by promoting the dephosphorylation of AKT in S473 residue [21,22]. However, scant knowledge exists regarding how PP2A Cα governs CD8^+^ T cells intrinsically and whether this regulation is essential for CD8^+^ T cell functionality. The use of T cell-specific deletion mice enabled further exploration of the role of PP2A in CD8^+^ T cells.

In this study, we used the pre-established *Ppp2ca*^fl/fl^/dLck-Cre mouse model to study the role of PP2A deletion on the effector function of CD8^+^ T cells during lymphocytic choriomeningitis virus (LCMV) infection and tumor challenge [23]. Our findings revealed that CD8^+^ T cells lacking catalytically active PP2A displayed compromised antiviral and antitumor efficacy. This conclusion was further corroborated by crossing *Ppp2ca*^fl/fl^ with CD8a-Cre mouse. Stimulation of CD8^+^ T cells in vitro also revealed that PP2A regulated cell activation in response to CD28 signaling. The augmentation of AKT S473 phosphorylation by PP2A thus serves as a new mechanism that enables CD8^+^ T cells to sustain proliferation and maintain a cytokine profile conducive to their effector function. Therefore, our discoveries uncover an unacknowledged function of PP2A in the activity of CD8^+^ T cells.

## Results

### PP2A deficiency leads to CD8^+^ T cell effector function impairment

**Fig. 1.**
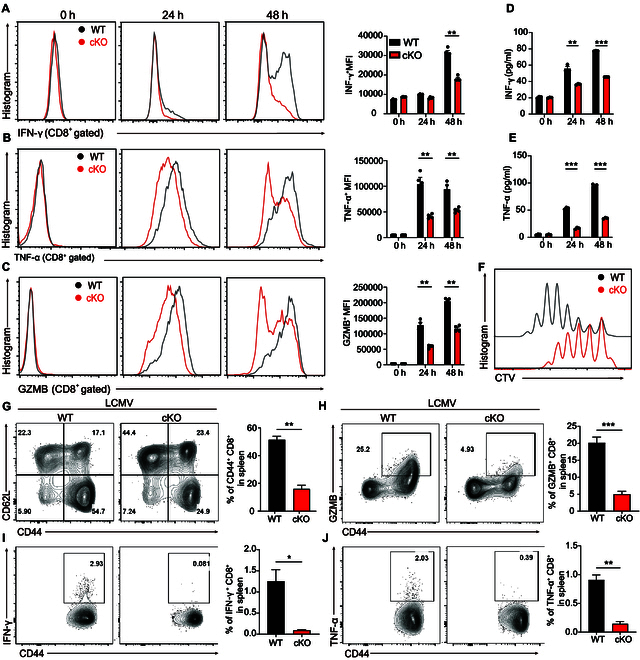
PP2A deficiency inhibits CD8^+^ T cell effector function. (A to C) WT or PP2A-deficient CD8^+^ T cells were stimulated with anti-CD3 (0.1 ng/μl) plus anti-CD28 (3 ng/μl) for the specified durations (0, 24, and 48 h). IFN-γ, TNF-α, and GZMB expression were measured by flow cytometry (intracellular cytokine staining). Representative images of IFN-γ, TNF-α, and GZMB intracellular staining and mean fluorescence intensity of the stained cells are presented. (D and E) Supernatant from (A) to (C) was harvested and assayed for IFN-γ and TNF-α by ELISA. (F) CTV-labeled naïve CD8^+^ lymphocytes were isolated from WT and *Ppp2ca*^fl/fl^/dLck^cre^ mice and stimulated for 3 d. Flow cytometry was used to assess the dilution of CTV on the CD8^+^ T cells. (G) Splenocytes from the LCMV-infected mice (5 d after infection) were isolated and gated on CD8^+^ T cells. Representative plots depicting the number of CD44^+^ CD8^+^ T cells (left) and their percentage (right) among the splenocytes of mice of different types (*n* = 3). (H to J) Isolated splenocytes from (G) were gated on CD8^+^ T cells. Representative plots (left) and the frequency (right) of GZMB^+^ CD44^+^ (H), IFN-γ^+^ CD44^+^ (I), and TNFα^+^ CD44^+^ (J) among CD8^+^ T cells are presented (*n* = 3). Mean ± SEM. *P < 0.05, **P < 0.01, ***P < 0.001, 2-tailed unpaired Student’s *t* test.

First, we used the T cell-specific PP2A Cα knockout (KO) mice (*Ppp2ca*^fl/fl^/dLck-Cre) to investigate the role of PP2A in peripheral CD8^+^ T cells [18]. Furthermore, the proportion of naïve/effector T cells in peripheral were also similar between wild-type (WT) and *Ppp2ca*^fl/fl^/dLck-Cre mice as we described in our previous story [18]. The normal maturation of peripheral lymphocytes and the targeted deletion of PP2A Cα protein in CD8^+^ T cells in these mice enabled additional exploration of the function of PP2A in CD8^+^ T cells [18]. Subsequently, we utilized these mice to test the effect of endogenous PP2A Cα in the T cell response. Upon activation in vitro, naïve CD8^+^ T cells (CD44^low^ CD62L^high^) from PP2A Cα-deficient mice exhibited decreased production of inflammatory cytokines like interferon-γ (IFN-γ), tumor necrosis factor-α (TNF-α), and granzyme B (GZMB) (Fig. 1A to E). PP2A Cα-deficient CD8^+^ T cells exhibited decreased cell proliferation capacity under identical stimulation conditions, as evidenced by CellTrace Violet (CTV) dilution assay (Fig. 1F).

We used LCMV Armstrong to induce acute infection and assayed the CD8^+^ T cell-mediated antiviral response to confirm the contribution of PP2A in CD8^+^ T cell function in vivo. At 5 d after infection, we observed that there were more CD8^+^ T cells in the spleens of WT mice compared to *Ppp2ca*^fl/fl^/dLck-Cre mice, evidenced by both frequency and absolute number. The proportion and cell number of effector cells (CD44^high^ CD62L^low^) were also significantly higher in WT mice (Fig. 1G and Fig. S1A). Furthermore, decreased frequency and cell numbers of GZMB producers, IFN-γ, and TNF-α-producing cells among CD8^+^ T cells were detected in cKO mice (Fig. 1H to J and Fig. S1B to D). As a result, there were higher viral titers in the *Ppp2ca*^fl/fl^/dLck-Cre mice compared with WT mice in both liver and spleen tissues, as shown in Fig. S1E and F. We then explored the impact of PP2A in antigen-specific CD8^+^ T cell response to infection. The percentage of antigen-specific CD8^+^ T cells recognizing *Listeria monocytogenes* (LM-OVA) were reduced in PP2A conditional KO (cKO) mice compared to control mice (Fig. S1G and I). Upon ex vivo restimulation with the OVA^257–264^ peptide, there were less cytokine-producing cells in PP2A cKO mice than in control mice (Fig. S1H and J). The above data suggest that the ablation of PP2A Cα results in reduced effector function of CD8^+^ T cells.

### Reduced antitumor response by PP2A-deficient CD8^+^ T cells

**Fig. 2.**
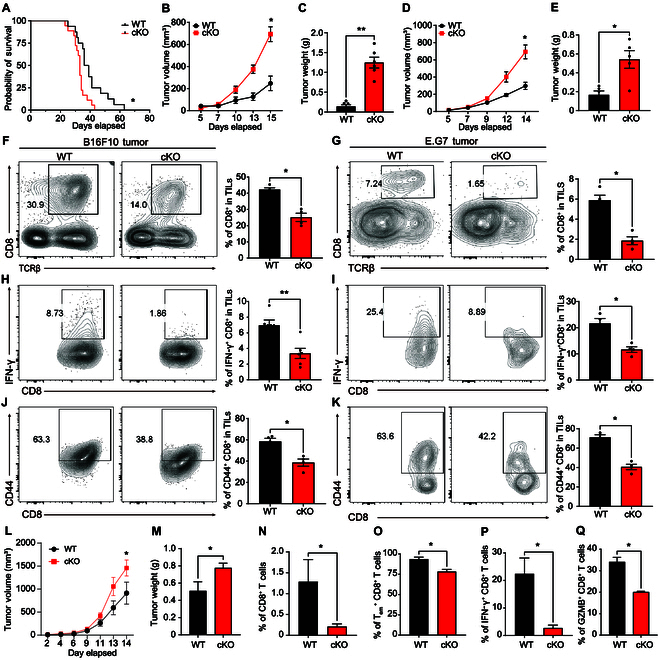
Reduced antitumor response by PP2A-deficient CD8^+^ T cells. (A to C) B16F10 tumor cells were intravenously injected to WT and *Ppp2ca*^fl/fl^/dLck^cre^ mice to stimulate lung metastasis model in vivo, and the survival rate was assessed (A). WT and *Ppp2ca*^fl/fl^/dLck^cre^ mice (aged 8 to 14 weeks) were subcutaneously implanted with 2 × 10^5^ B16F10 cells. Tumor volume (B) and weight (C) were assessed on day 15 after implantation, coinciding with the euthanasia of mice (*n* = 6). (D and E) WT and *Ppp2ca*^fl/fl^/dLck^cre^ mice (aged 8 to 14 weeks) received 5 × 10^5^ E.G7 cells subcutaneously. Tumor volume (D) was measured during the next 14 d. Animals were sacrificed on day 14, and their tumor weight (E) was measured (*n* = 5). (F and G) Mice challenged with tumor cells were sacrificed on day 14, and flow cytometry was used to calculate the proportion of CD8^+^ T cells among TILs from mice with B16F10 tumors (F) and E.G7 tumors (G). Representative flow cytometry plots (left) and summary statistics (right) are shown. (H and I) Mice challenged with tumor cells were sacrificed on day 14, and the proportion of IFN-γ^+^ CD8^+^ T among TILs from B16F10 tumor-bearing (H) and E.G7 tumor-bearing (I) mice was detected by flow cytometry. Representative flow cytometry plots (left) and summary statistics (right) are shown. (J and K) Mice challenged with tumor cells were sacrificed on day 14, and the proportion of CD44^high^ CD8^+^ T among TILs from B16F10 tumor-bearing (J) and E.G7 tumor-bearing (K) was detected by flow cytometry. Representative flow cytometry plots (left) and summary statistics (right) are shown. Each graph represents data from 3 independently repeated experiments (*n* = 4 to 6) (F to K). (L to Q) Naïve WT or PP2A KO CD8^+^ T cells (1 × 10^6^) were isolated and adoptively transferred into *Rag1*^−/−^ mice, and followed by E.G7 tumor challenge. Tumor volume (L) and weights (M) of mice were assessed 14 d later. The proportion of total CD8^+^ T cells (N), effector memory T cells (O), IFN-γ^+^ CD8^+^ T cells (P), and GZMB^+^ CD8^+^ T cells (Q) among TILs of recipient *Rag1*^−/−^ mice was analyzed by FACS. Related to Fig. S2H to J. Mean ± SEM; *P < 0.05, **P < 0.01, 2-tailed unpaired Student’s *t* test for (B) to (Q) and Kaplan–Meier method for mouse survival (A)

We further examined the effect of PP2A deficiency on CD8^+^ T cells in antitumor response by 2 distinct tumor models. PP2A-deficient mice showed a significantly reduced survival rate compared with the WT group after being intravenously injected with B16F10 cells (Fig. 2A). When grafted under the skin, B16F10 cells grew into significantly bigger tumors in *Ppp2ca*^fl/fl^/dLck-Cre mice (Fig. 2B and C). Similar results were observed in another E.G7 lymphoma tumor model, indicating that the requirement of PP2A in CD8^+^ T cell-mediated antitumor response is a general fact (Fig. 2D and E).

Subsequently, we examined TILs to elucidate the disparities in CD8^+^ T cell-driven antitumor responses. Initially, a reduction of CD8^+^ T cell infiltration was observed in the tumors of PP2A cKO mice, as shown by staining with TCR-β and CD8a (Fig. 2F and G). Tumor-infiltrating CD8^+^ T cells from *Ppp2ca*^fl/fl^/dLck^cre^ mice exhibited reduced CD44^+^ cell population and markedly decreased IFN-γ production (Fig. 2H to K). Furthermore, deficiency of PP2A in CD8^+^ T cells resulted in impaired function, as evidenced by the defective production of TNF-α and GZMB (Fig. S2A and B). When we stained CD8^+^ T cells along with in situ TUNEL (terminal deoxynucleotidyl transferase–mediated deoxyuridine triphosphate nick end labeling) staining, we observed impaired infiltration of tumors with CD8^+^ T cells and a decreased number of TUNEL-positive tumor cells in tumors from *Ppp2ca*^fl/fl^/dLck^cre^ mice (Fig. S2C). In accordance with this finding, we observed that the expression of exhaustion markers including PD-1 and TIGIT in CD8^+^ TILs from *Ppp2ca*^fl/fl^/dLck^cre^ mice was elevated (Fig. S2D and E). Conversely, there was no significant difference in CD4^+^ T cells between WT and *Ppp2ca*^fl/fl^/dLck^cre^ mice (Fig. S2F). Collectively, these findings indicate that PP2A affects the cytotoxic and antitumor properties of CD8^+^ T cells.

To get rid of the complex effect from other T cells and precisely ascertain the role of PP2A in CD8^+^ T cells [18–20], naïve CD8^+^ T cells were isolated from WT and *Ppp2ca*^fl/fl^/dLck^cre^ mice and subsequently adoptively transferred into immunodeficient *Rag1*^−/−^ recipient mice, which were then subjected to a tumor challenge (Fig. S2G). Mice that received *Ppp2ca*^fl/fl^/dLck^cre^ CD8^+^ T cells exhibited increased tumor volume compared with the WT group (Fig. 2L to N). Again, PP2A-deficient CD8^+^ T cells exhibited reduced tumor infiltration and fewer cells expressed IFN-γ, TNF-α, and GZMB (Fig. 2O to Q and Fig. S2H to J). The expression of exhaustion marker TIM3 was also increased in CD8^+^ T cells lacking PP2A when compared with WT cells (Fig. S2K). All these data indicate that PP2A regulates the ability of CD8^+^ T cells to exert their functions, in particular, during the antitumor immune response.

### Specific deletion of PP2A in CD8^+^ T cells does not change CD8^+^ T cell homeostasis but attenuates their antitumor activity

PP2A is known to play crucial roles in multiple cellular process, and the alteration in T cell homeostasis could affect the effector functions of T cells. We crossed *Ppp2ca*^fl/fl^ mice with CD8a^cre^ mice (*Ppp2ca*^fl/fl^/CD8a^cre^) to conditionally delete PP2A Cα only in peripheral CD8^+^ T cells to examine the general effects of PP2A KO on the homeostasis of CD8^+^ T cells (Fig. S3A). We observed that WT and *Ppp2ca*^fl/fl^/CD8a^cre^ showed comparable cellularity in the spleen, lymph nodes, mucocutaneous lymph node, and peripheral blood (Fig. S3B to D). We conducted an additional analysis of the naïve and memory T cell subsets, and found an identical cell type distribution pattern (Fig. S3E to H). These data showed that PP2A is dispensable for CD8^+^ T cell homeostasis, thus allowing us to further investigate the impact of PP2A on the CD8^+^ T cell effector functions.

**Fig. 3.**
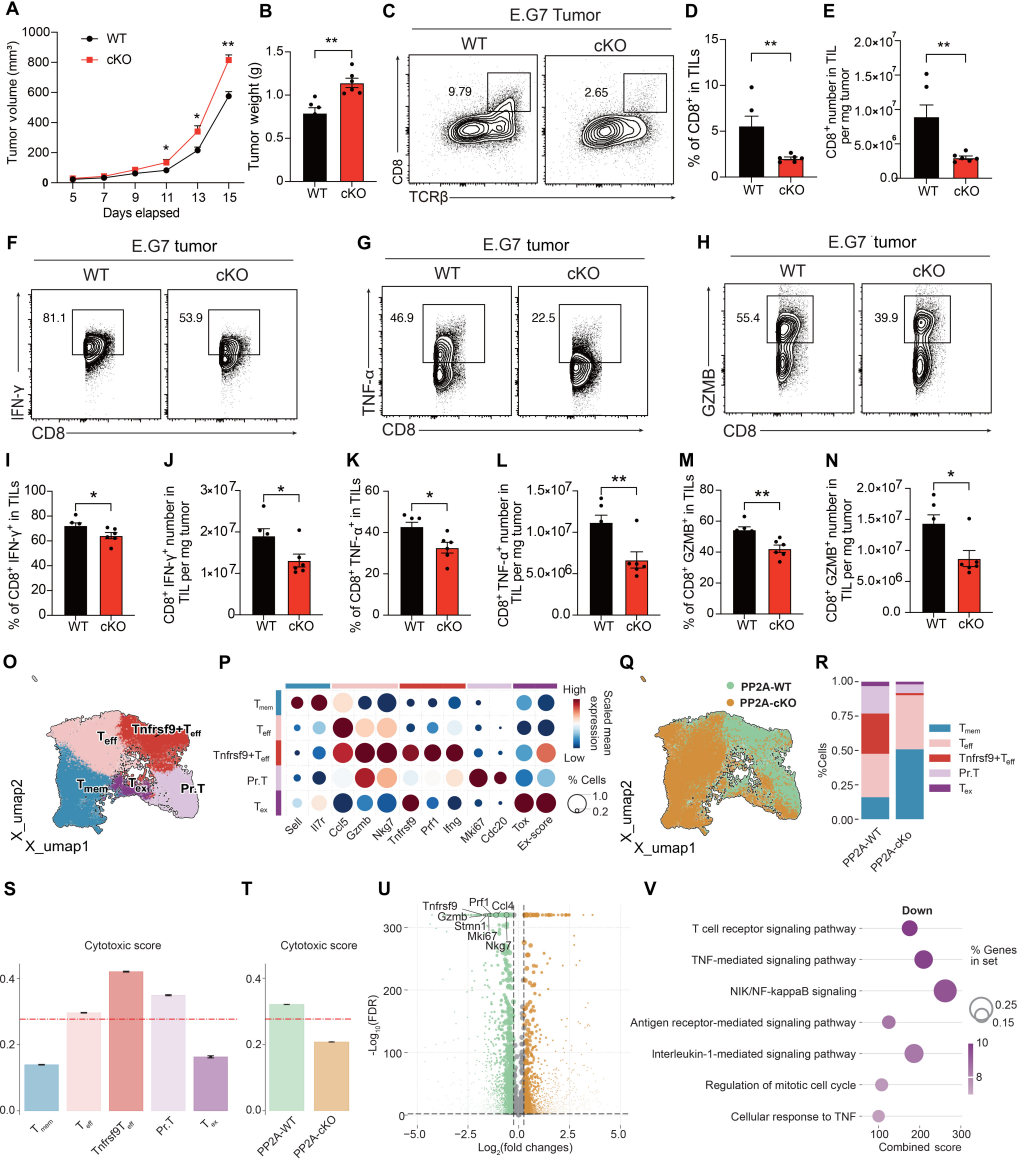
Specific deletion of PP2A in CD8^+^ T cells attenuates its antitumor properties. (A and B) WT and *Ppp2ca*^fl/fl^/CD8a^cre^ mice (8 to 14 weeks old) were subcutaneously injected with 5 × 10^5^ E.G7 cancer cells and euthanized at 14 d later (*n* = 5). Tumor volume (A) and weight (B) were assessed on day 14 following engraftment, coinciding with the euthanasia of the mice (*n* = 6). (C to E) The phenotype of TILs (C) and the percentage (D) and cell number count (E) of CD8^+^ T cells among E.G7 TILs were assessed using flow cytometry (*n* = 6). (F to N) Representative flow cytometry plots and bar plots showing the percentage and total cell count of CD8^+^ IFN-γ^+^ T cells (F, I, and J), CD8^+^ TNFα^+^ T cells (G, K, and L), and CD8^+^ GZMB^+^ T cells (H, M, and N) within TILs (*n* = 6). (O to V) CD8^+^ TILs were isolated for scRNA-seq, and 5 clusters of CD8^+^ T cells were identified (O) and characterized by their signature genes (P). UMAP plot showing the difference between WT and cKO CD8^+^ T cells (Q). Proportions of different cell types (R). The cytotoxic score for the 5 CD8^+^ T cell subsets (S). The cytotoxic score between WT and cKO CD8^+^ T cells (T). Differentially expressed genes between WT and cKO CD8^+^ T cells by DEG analysis (U). GSEA network analysis of down-regulated pathways in PP2A cKO CD8^+^ TILs (V). Mean ± SEM; *P < 0.05, **P < 0.01, 2-tailed unpaired Student’s *t* test.

We again performed a subcutaneous E.G7 tumor model. The implanted tumor cells displayed markedly increased growing scales in *Ppp2ca*^fl/fl^/CD8a^cre^ mice than those in WT mice, as indicated by larger tumor volume and increased tumor weight (Fig. 3A and B). The frequency and quantity of CD8^+^ T cells were notably reduced in *Ppp2ca*^fl/fl^/CD8a^cre^ mice in TILs (Fig. 3C to E). In addition, the PP2A-deficient CD8^+^ T cells that produced much less IFN-γ, TNF-α, and GZMB (Fig. 3F to N) exhaustion markers such as PD-1 and TIGIT were found to be increased in CD8^+^ TILs from *Ppp2ca*^fl/fl^/Cd8a^cre^ mice, mirroring the phenotype observed in *Ppp2ca*^fl/fl^/dLck^cre^ mice (Fig. S3I and J).

To examine the role of PP2A in regulating the phenotype of CD8^+^ TIL, we performed single-cell RNA sequencing (RNA-seq) on CD8^+^ T cells isolated from E.G7 tumor. Unsupervised clustering identified 5 clusters of CD8^+^ TILs, characterized by their signature genes: memory T cells (T_mem_), effector T cells (T_eff_), Tnfrsf9^+^ effector T cells (Tnfrsf9^+^ T_eff_), proliferating T cells (Pr. T), and exhausted T cells (T_ex_) (Fig. 3O and P). Notably, Tnfrsf9^+^ T_eff_ was featured with a higher cytotoxic score and showed elevated expression of cytotoxic genes, such as Gzmb and Prf1 (Fig. 3P and Q). PP2A deficiency resulted in a reduced frequency of Tnfrsf9^+^ T_eff_ and Pr. T, illustrating the impaired cytotoxicity and proliferation ability of CD8^+^ TIL, indicating impaired cytotoxicity and proliferative capacity of CD8^+^ TILs (Fig. 3R and S). This observation aligned with the reduced cytotoxic score of CD8^+^ TILs in PP2A-deficient mice (Fig. 3T). Additionally, differentially expressed gene (DEG) analysis showed that PP2A KO led to down-regulation of cytotoxic and proliferation-associated genes, including *Gzmb, Prf1, Nkg7, Tnfrsf9, Stmn1*, and *Mki67* (Fig. 3U). Gene ontology enrichment analysis demonstrated that pathways involved in T cell receptor (TCR) signaling, TNF, nuclear factor κB (NF-κB), and cell cycle progression were significantly down-regulated in PP2A-cKO mice (Fig. 3V). These findings collectively underscore the critical role of PP2A in maintaining the cytotoxic and proliferative potential of CD8^+^ TILs, suggesting its importance in antitumor immune responses.

Altogether, the consistent CD8^+^ T cell phenotype observed in *Ppp2ca*^fl/fl^/Cd8a^cre^ and *Ppp2ca*^fl/fl^/dLck^cre^ mice underscores the specific, cell-intrinsic role of PP2A in the regulation of CD8^+^ T cell effector function.

### PP2A deficiency impedes CD28 signaling

**Fig. 4.**
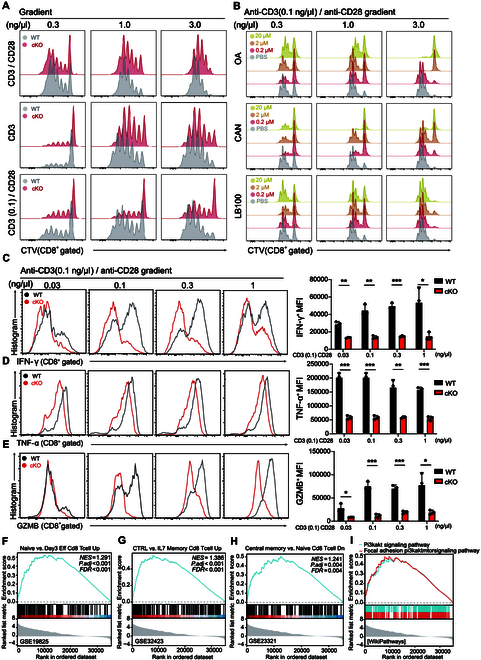
PP2A deficiency impedes CD28 signaling. (A) Naïve WT and PP2A-deficient CD8^+^ T cells were labeled with CTV and subsequently stimulated with varying concentrations of CD3 and/or CD28 antibodies (0.3 to 3.0 ng/μl, top), anti-CD3 alone (0.3 to 3.0 ng/μl, middle), or anti-CD3 (0.1 ng/μl) + anti-CD28 (0.3 to 3.0 ng/μl, bottom) for a duration of 72 h. (B) Naïve WT CD8^+^ T cells were stained with CTV and stimulated with anti-CD3 (0.1 ng/μl) plus anti-CD28 (0.3 to 3 ng/μl) for 72 h, and cells were treated with a gradient of concentrations (0, 0.2, 2, and 20 μM) of 3 PP2A inhibitors—cantharidin (CAN, top), okadaic acid (OA, middle), and LB100 (bottom)—in the meantime. (C to E) Naïve CD8^+^ T cells were isolated and stimulated with plate-bound anti-CD3 (0.1 ng/μl) plus gradient anti-CD28 (0.03 to 1 ng/μl) for 48 h. Expression profile of IFN-γ, TNF-α, and GZMB was analyzed by flow cytometry. Representative flow cytometry plots (left) and mean fluorescence intensity (MFI) statistics (right) are shown (*n* = 3). (F to I) Naïve CD8^+^ T cells from WT and *Ppp2ca*^fl/fl^/CD8a^cre^ mice were stimulated with anti-CD3 (0.1 ng/μl) plus anti-CD28 (3 ng/μl) antibodies for 1 h, and then bulk RNA-seq was performed. Gene Set Enrichment Analysis (GSEA) revealed enrichment of a gene set associated with effector and memory signatures (F to H). The PI3K–AKT signaling pathway gene set exhibited enrichment in the PP2A WT group (I). *n* = 3. Mean ± SEM; *P < 0.05, **P < 0.01, ***P < 0.001, 2-tailed unpaired Student’s *t* test.

To gain insight into how specific deletion of PP2A in T cells leads to inadequate CD8^+^ T cell-mediated antitumor responses, we first determine whether PP2A deficiency affects T cell activation. To our surprise, the deletion of PP2A did not affect the induction of CD69 expression and IL-2 production, which are the classical T cell activation tests in vitro, by optimal anti-CD3 plus anti-CD28 stimulation (Fig. S4A and B). Similarly, TCR-induced CD8^+^ T cell proliferation was unaltered with or without CD28 costimulation (Fig. 4A, top 2 rows). Based on the fact that the CD28 costimulation effect could only be observed in suboptimal TCR stimulation conditions in vitro [24], we thus limited the concentration of anti-CD3 (0.1 ng/μl) and added gradient of anti-CD28 monoclonal antibodies in this in vitro stimulation assay. Under this condition, the difference of cell proliferation between WT and PP2A cKO cells became obvious (Fig. 4A, bottom line). When PP2A inhibitors—cantharidin (CAN), okadaic acid (OA), and LB100—were applied to culture medium, they also specifically inhibit CD28-dependent CD8^+^ T cell proliferation under the limited concentration of anti-CD3 concentration (0.1 ng/μl) with the gradients of CD28 monoclonal antibody doses (Fig. 4B). At the same time, we found that neither PP2A KO nor the use of its inhibitor affected cell apoptosis (Fig. S4C and D). Consistent with the previous observation (Fig. 1A), the attenuation of effector function was more pronounced in those CD8^+^ T cells lacking PP2A under this condition (Fig. 4C to E). To further reveal that the altered signal resulted in impaired activation in PP2A cKO CD8^+^ T cells, bulk RNA-seq analysis was then implemented on anti-CD3 (0.1 ng/μl) plus anti-CD28 (3 ng/μl)-treated naïve CD8^+^ T cells. Activated WT CD8^+^ T cells exhibited a greater enrichment of signature genes associated with effector and central memory CD8^+^ T cells compared to the PP2A KO group (Fig. 4F to H). As a major target of CD28 costimulation signal, the phosphatidylinositol 3-kinase (PI3K)–AKT signaling pathway was also enriched more prominently in WT CD8^+^ T cells (Fig. 4I). The compromised effector function in CD8^+^ T cells lacking PP2A is also reflected by the differential gene expression in WT and cKO groups (Fig. S4E to G) [25–28]. All these data demonstrate that PP2A KO leads to the attenuation of CD28 signaling.

### PP2A promotes CD8 effector function by augmenting AKT phosphorylation

**Fig. 5.**
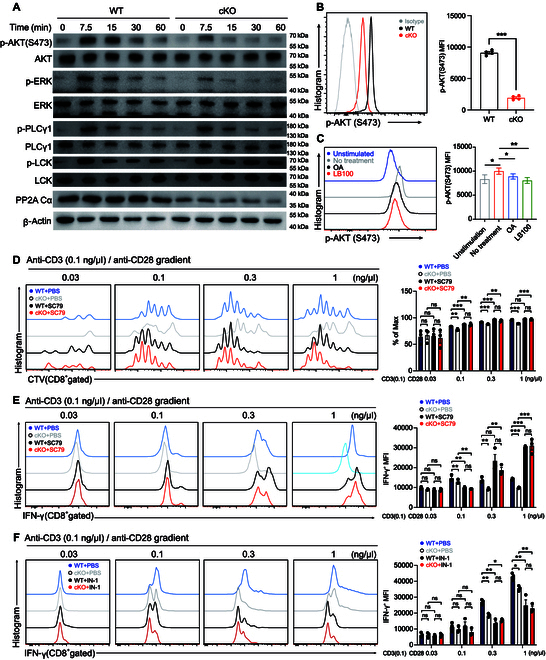
PP2A promotes CD8 effector function by augmenting AKT phosphorylation. (A) Naïve CD8^+^ T cells were stimulated with anti-CD3 (0.5 ng/μl) plus anti-CD28 (2 ng/μl) for the indicated time. Subsequently, the cells were used for immunoblotting to detect total and phosphorylated AKT, ERK, PLCG1, and LCK. (B) Naïve CD8^+^ T cells were stimulated as described above for 1 h. Flow cytometry was used to detect the level of pAKT (S473) in CD8^+^ T cells. Representative plots are shown on the left, and statistics of MFI is shown on the right (*n* = 4). (C) Naïve CD8^+^ T cells were either untreated or pretreated with OA and LB100 (left). Subsequently, cells were stimulated as described above. pAKT levels in CD8^+^ T cells were then assessed by flow cytometry. MFI of pAKT was quantified (right). Blue line, unstimulated control; gray line, no treatment; black line, OA treatment; red line, LB100 treatment. (D) WT and PP2A-deficient naïve CD8^+^ T cells were stained with CTV and stimulated with anti-CD3 (0.1 ng/μl) plus anti-CD28 (in gradient 0.03 to 1 ng/μl) in the presence of PBS or SC79 for 72 h. Proliferation was assessed by flow cytometry by the CTV dilution. Representative plots are shown on the left, and statistics is shown on the right (*n* = 3). (E) Expression of IFN-γ from the cells stimulated in the abovementioned conditions was assessed by intracellular cytokine staining. Representative plots (left) and statistics of MFI (right) are presented (*n* = 3). (F) PP2A-deleted and contrast naïve CD8^+^ T cells were stimulated with 0.1 ng/μl CD3 antibody plus anti-CD28 (in gradient 0.03 to 1 ng/μl) in the presence of PBS or IN-1 for 72 h. Expression profile of IFN-γ was assessed by intracellular cytokine staining. Representative plots (left) and statistics of MFI (right) are presented (*n* = 3). Mean ± SEM; *P < 0.05, **P < 0.01, ***P < 0.001, 2-tailed unpaired Student’s *t* test.

AKT and extracellular signal-regulated kinase (ERK) are pivotal components of CD28 and TCR activation signaling cascades. In the previous step, we observed that CD8^+^ T cells that lack functional PP2A (cKO or after the pharmacological inhibition) demonstrated defects during the stimulation in vitro at low levels of TCR and CD28 stimulation (Fig. 4). Thus, we next assessed the signaling activation status downstream of TCR/CD28 stimulation. Western blot analysis revealed down-regulated pAKT (S473) and pERK (T202/T204) in PP2A-deficient cells (Fig. 5A). Furthermore, there were no significant changes observed in the phosphorylation of PLCγ1 and LCK, the downstream components of TCR signaling (Fig. 5A). More importantly, the difference in AKT phosphorylation between WT and cKO cells was evident only in the presence of anti-CD28 costimulation (Fig. 5A and Fig. S5A). The difference observed in Western blot could be further confirmed by flow cytometry analysis (Fig. 5B). Additionally, treatment with the PP2A inhibitor replicated the phenotype (reduced Ser^473^ AKT phosphorylation) observed in PP2A cKO cells upon activation with stimulation (Fig. 5C).

To determine whether reduced AKT phosphorylation accounts for the impaired CD8^+^ T cell effector function upon PP2A deficiency, we applied AKT agonist SC79 to *Ppp2ca*^fl/fl^/Cd8a^cre^ CD8^+^ T cell and examined the cellular function [29]. We conducted in vitro assays to examine the proliferation of naïve CD8^+^ T cells and observed that treatment with SC79 significantly enhanced CD8^+^ T cell division in the WT group. Furthermore, SC79 treatment successfully reversed the proliferation defects observed in PP2A-deficient CD8^+^ T cells, thereby highlighting the potential role of the PP2A-AKT axis in modulating T cell proliferation (Fig. 5D). SC79 also restored the production of IFN-γ, TNF-α, and GZMB in PP2A-deficient CD8^+^ T cells at various concentrations of anti-CD28 stimulation. This effect was observed at both 24 and 48 h after stimulation, indicating a robust and sustained response (Fig. 5E and Fig. S5B to D). In contrast, the inhibitor of AKT IN-1 suppressed cytokine production in WT CD8^+^ T cells (Fig. 5F and Fig. S5E and F) [30]. Notably, AKT IN-1-induced cytokine production inhibition did not occur in PP2A-deficient CD8^+^ T cells (Fig. 5F and Fig. S5E and F). In summary, the aforementioned results validate that diminished AKT phosphorylation is responsible for the compromised effector function observed in CD8^+^ T cells with PP2A deficiency.

### The expression and targeting of PP2A in tumor

**Fig. 6.**
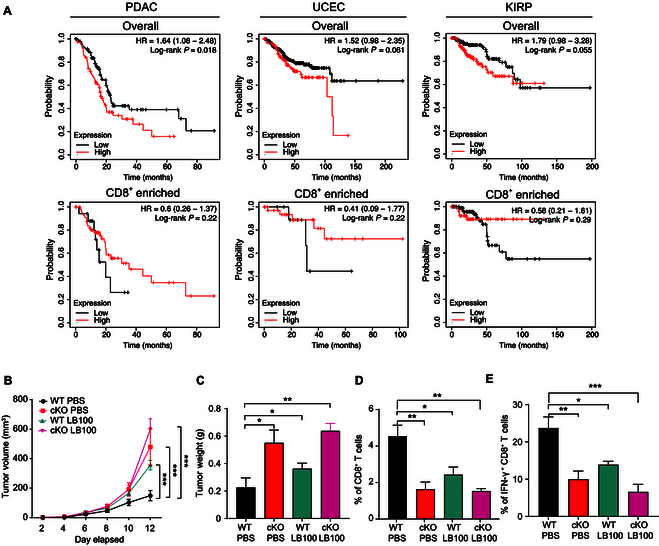
The expression and targeting of PP2A in tumor. (A) Correlation of PP2A expression with the overall survival of pancreatic cancer, uterine corpus endometrial carcinoma, or kidney renal papillary cell carcinoma for the whole tumor (top). The correlation of PP2A expression with enriched CD8^+^ T cell and prognoses in pancreatic cancer, uterine corpus endometrial carcinoma, or kidney renal papillary cell carcinoma. Data were obtained from KMplot.com. (B and C) WT and *Ppp2ca*^fl/fl^/dLck^cre^ mice (aged 8 to 14 weeks) were subcutaneously implanted with 5 × 10^5^ E.G7 cancer cells, treated with LB100 or PBS, and sacrificed on day 14 after implantation. Tumor volume (A) and weight (B) were assessed as described above (*n* = 4). (D and E) Flow cytometric analysis of the percentage of CD8^+^ T cells (D) or IFN-γ^+^ CD8^+^ T cells (E) among E.G7 TILs (*n* = 4). Related to Fig. S6B and C. Mean ± SEM; *P < 0.05, **P < 0.01, ***P < 0.001, 2-tailed unpaired Student’s *t* test.

To reveal the clinical relevance of our finding in mouse model, we next interrogated expression data of PP2A in human cancer tissues from the The Cancer Genome Atlas (TCGA) database. At the overall gene expression level, we could not find a definitive correlation between PP2A and disease prognosis. In some tumors, low levels of PP2A were associated with poor overall survival, while in some other tumors, the opposite trend was observed, or there was no correlation at all (Fig. 6A and Fig. S6A). However, when we specifically restricted the expression of PP2A in CD8^+^ TILs, we observed a positive correlation between high levels of PP2A expression in these cells and improved patient prognosis. This correlation was independent of the overall expression levels of PP2A in the tumor, suggesting a critical role for PP2A within CD8^+^ TILs in influencing clinical outcomes, thus indicating that the abundance of PP2A in CD8^+^ T cells is beneficial in controlling tumor development (Fig. 6A).

In this study, we observed that the targeted ablation of PP2A in CD8^+^ T cells significantly compromised the antitumor immune response in mice. This impairment was characterized by a reduction in effector functions, diminished cytokine production, and decreased infiltration of CD8^+^ T cells into the tumor microenvironment, ultimately leading to an ineffective tumor suppression. In contrast, some other papers have suggested that the use of PP2A inhibitors enhances immune responses [31,32]. Therefore, we utilized LB100 to further explore the potential of targeting PP2A in cancer therapy. E.G7 cells were subcutaneously injected into the dorsal region of the mice, followed by treatment with LB100. Tumors in the LB100-treated cohort demonstrated a marked increase in tumor size relative to those in the control group receiving vehicle treatment (Fig. 6B and C). While conflicting with some of the studies mentioned above, this result aligns with some other reports and is supported by the phenotypes observed in PP2A cKO mice [31–34]. We also observed a certain degree of tumor promotion in the PP2A cKO mouse group treated with the inhibitor, suggesting that inhibition of PP2A on tumor promotion is not limited to CD8^+^ T cells (Fig. 6B to E and Fig. S6B and C). Given that PP2A inactivation has been identified as a critical step in malignant transformation, PP2A agonists were expected to make double hits on both tumor cells and CD8^+^ T cells [35–37]. In contrast, the wide-ranging effects of PP2A inhibitors underscore the necessity for increased caution when contemplating their application in cancer therapy [32,38,39].

## Discussion

Utilizing T cell-specific KO mice in the peripheral immune system, we elucidated the essential regulatory role of intrinsic PP2A in CD8^+^ T cell effector functions. Our findings highlight its critical involvement in modulating immune responses against both tumors and infections, affirming PP2A’s significance in T cell-mediated immunity. Similarly, PP2A inhibitors also lead to T cell lesion and tumor progression. By modulating the phosphorylation of AKT at the Ser^473^ site, our study revealed an unanticipated role of PP2A in CD8^+^ T cells. This mechanism is distinct from the regulatory pathways previously observed in CD4^+^ T cells, underscoring the unique function of PP2A in enhancing the effector capabilities of CD8^+^ T cells. This underscores the pivotal role of AKT signaling in the functional integrity of CD8^+^ T cells and highlights the impact of PP2A on modulating this pathway. Consistent with our previous findings, PP2A is dispensable for TCR signaling, but is involved in modulating secondary signals (costimulation) in CD8^+^ T cells and cytokine stimulation [transforming growth factor-β (TGF-β)] in CD4^+^ T cells, which are crucial for the development of their effector functions [15,18,19].

Concurrent with our investigation, another research group has recently published a preprint manuscript proposing the pivotal role of PP2A in maintaining homeostasis among peripheral CD8^+^ T cells [40]. Using the CD4-Cre mouse model, they observed that CD8^+^ T cells deficient in PP2A Cα exhibited attenuated proliferation and decreased viability. However, based on our previous research on the use of various T cell-specific Cre strains, CD4-Cre-mediated gene KO often leads to changes in late-stage thymic development, leading to altered output of T cells to the periphery. Based on the data we obtained from *Ppp2ca*^fl/fl^/dLck^cre^ and *Ppp2ca*^fl/fl^/Cd8a^cre^ mice, where CD8^+^ T cell homeostasis remained unaffected, we speculate that the primary reasons for the homeostasis alterations observed in *Ppp2ca*^fl/fl^/CD4^Cre^ mice originate from thymic development change [15].

CD28 costimulation is crucial for initiating T cell activation and facilitating the maturation of T cell effector functions [41]. Chuang et al. [42] previously proposed that suppression of PP2A activity, achieved through inhibitor OA treatment or expression of a dominant-negative mutant in Jurkat cells, a human T cell line, may potentiate T cell activation following CD28 engagement. PP2A was also considered one of the key regulatory factors in CTLA-4 signal transduction. The A subunit of PP2A binds to the lysine-rich motif situated in the juxtamembrane region of the cytoplasmic tail of human CTLA-4, thereby enhancing PP2A activity and suppressing T cell activation and proliferation. This suggests that PP2A acts as a crucial regulator in the immunosuppressive effects mediated by CTLA-4 [43]. Teft et al. [11] reported that the PP2A Cα subunit binds to the tyrosine residue of CTLA-4 situated within the YVKM motif at position 165. Nonetheless, uncertainties persist regarding the in vivo occurrence of these interactions within T cells and their potential functional implications. Our study indicates that PP2A plays a crucial role in enhancing CD28 signaling regulation. Our findings demonstrate that deficiency in PP2A results in diminished proliferative capacity and reduced cytokine production in CD8^+^ T cells following stimulation with suboptimal concentrations of anti-CD3 and anti-CD28. The parallel results were obtained when pharmacologically inhibiting PP2A by treatment with OA, CAN, and LB100. It is worth noting that many previous conclusions have been based on biochemical and cellular studies. Our research elucidates the genetic perspective on PP2A’s role in regulating CD8^+^ T cell activation signals, aiming to better approximate its in vivo functionality. However, further investigation into its detailed mechanisms of action will be necessary.

Some of the studies discussed above suggest that targeting PP2A may enhance immune responses. Zhuang and colleagues [31] demonstrated that the synergistic application of the PP2A inhibitor LB100 alongside PD-1 blockade augmented mice’s antitumor efficacy. This effect was concomitant with heightened lymphocyte activation. Lu and colleagues [44] discovered that mice deficient in macrophage PP2A exhibited reduced tumor progression through modulation of the PP2A/STRN4-YAP/TAZ axis. On the other hand, PP2A inactivation also has been described as the vital step in malignant transformation [32,45,46]. Several groups have explored the possibility of simultaneously reactivating PP2A and inhibiting kinases as a potential therapy to mitigate alterations in tumor suppressor genes and oncogenes implicated in cancer formation [32,36,37,47–49]. These results suggest that targeting PP2A in tumor therapy may generate different results from the combined effects on multiple cell types. Through genetic methodologies, our results reveal the advantageous function of PP2A in CD8^+^ T cells, as the deletion of PP2A impairs the effector function of CD8^+^ T cells. Taken together, these findings suggest that when considering the use of PP2A inhibitors for immunotherapy, it is crucial to account for their varying effects across different cell types and assess their potential therapeutic benefits.

In summary, our study confirmed that PP2A-deficient CD8^+^ T cells have altered phosphorylation of AKT at Ser^473^ downstream of CD28 and diminished proliferation, activation, and cytokine expression. AKT has been reported to play a predominant role in peripheral CD8^+^ T cells by promoting the activation and subsequent differentiation into short-lived effective cells, which is consistent with our results [50,51]. Although we have established the modulation and significance of AKT phosphorylation by PP2A in CD8^+^ T cells, a limitation of this study lies in its inability to discern the specific substrate of PP2A responsible for direct dephosphorylation leading to AKT activation, necessitating further investigation. In addition, we observed elevated expression of exhaustion-related markers on tumor-infiltrating CD8^+^ T cells in PP2A cKO mice. This phenomenon may be linked to dysregulated AKT signaling in PP2A-deficient CD8^+^ T cells, or PP2A might regulate CD8^+^ T cell exhaustion through alternative mechanisms, influencing their effector function. Furthermore, PP2A exerts different functions through distinct substrates, such as the PP2A B subunit, and studies on the role of this subunit in our system are still lacking. While earlier reports have indicated that PP2A B55β regulates apoptosis in CD8^+^ T cells by promoting AKT dephosphorylation, which contrasts with the findings of this study, the intricate regulatory mechanisms of phosphatases like PP2A, with their broad substrate specificity and transient enzyme–substrate interactions, may account for the discrepancy. Therefore, these conclusions are not necessarily conflicting [21,22]. Given that the activity of phosphatase PP2A is essential for T cell activation, it is reasonable to suggest that PP2A may play a role in initiating CD28 signaling to control the function of CD8^+^ T cells [9,52].

## Materials and Methods

### Mice and cell lines

*Ppp2ca* floxed mice used in these experiments were described by Zheng et al. [15]. Mice with dLck-Cre and CD8a-Cre were purchased from The Jackson Laboratory. *Ppp2ca* KO was confirmed by genotyping as described before [18]. Distal Lck-Cre and CD8a-Cre mouse genotype identification refers to Jackson’s website [53,54]. All mice were housed under specific pathogen-free (SPF) conditions. Cell lines for B16F10 mouse melanoma and E.G7-OVA mouse melanoma were provided by C. Xu and D. Chen, respectively.

### Reagents

RPMI 1640 medium (Gibco), fetal bovine serum (Gibco), sodium pyruvate (Solarbio), Hepes (Solarbio), penicillin–streptomycin liquid (Solarbio), and β-mercaptoethanol (Solarbio) were used for mouse primary T cell culture. Supernatants from cell cultures were collected, and the concentrations of IFN-γ and TNF-α were measured by enzyme-linked immunosorbent assay (ELISA) (all from Thermo Fisher Scientific). Antibodies for fluorescence-activated cell sorting (FACS) or immunoblotting were described in Supplementary Materials and Methods. 

### Immunoblot to assess cell signaling

To detect TCR/CD28 cell signaling, 1 × 10^6^ naïve CD8^+^ T cells were resuspended in 100 μl of phosphate-buffered saline (PBS). Then, biotin-CD3 and/or biotin-CD28 were added at the corresponding concentrations. After that, streptavidin (SA) (Invitrogen) was added at 25 μg/ml. Cells were mixed thoroughly and incubated at 37 °C for the indicated periods of time. Once the stimulation was complete, the cells were washed twice with prechilled PBS. The composition of each reagent and detailed methods for Western blot were as previously described [55].

### In vivo animal model

WT and PP2A cKO mice were injected intraperitoneally with LCMV Armstrong [2 × 10^5^ plaque-forming units (PFU)/mice]. Mice were sacrificed 5 d after the LCMV challenge, and splenocytes were harvested for the evaluation of immune response alterations and cytokine expression in both WT and PP2A cKO mice.

*L. monocytogenes* [5 × 10^5^ colony-forming units (CFUs)] expressing the chicken ovalbumin (LM-OVA) were intraperitoneally injected into mice, and mice were sacrificed on day 7 after infection. Whole blood and splenocytes were isolated to measure antigen-specific CD8^+^ T cell response.

Tumor cells were mixed with a PBS solution that included 50% Matrigel from Corning Inc. to facilitate tumor implantation. Mice aged 6 to 8 weeks were injected with 2 × 10^5^ or 5 × 10^5^ B16F10 mouse melanoma cells or 5 × 10^5^ E.G7-OVA mouse lymphoma cells subcutaneously on the right flank. Detailed description was in Supplementary Materials and Methods.

### Single-cell RNA-seq library preparation and sequencing

Isolated CD8^+^ TILs were loaded into microfluidic chip of Chip A Single Cell Kit v2.1 [MobiDrop (Zhejiang) Co. Ltd., catalog no. S050100301] to generate droplets with MobiNova-100 [MobiDrop (Zhejiang) Co. Ltd., catalog no. A1A40001]. After encapsulation, droplets suffer light cut by MobiNovaSP-100 [MobiDrop (Zhejiang) Co. Ltd., catalog no. A2A40001], while oligos diffuse into reaction mix. The mRNAs were captured by cell barcodes with cDNA amplification in droplets. Following reverse transcription, cDNAs with barcodes were amplified, and a library was constructed using the High Throughput Single-Cell 3’ Transcriptome Kit v2.1 [MobiDrop (Zhejiang) Co. Ltd., catalog no. S050200301] and the 3’ Dual Index Kit [MobiDrop (Zhejiang) Co. Ltd., catalog no. S050300301]. Library sequencing was performed on Illumina NovaSeq X Plus platform with 150–base pair paired-end reads (Repugene Technology, Hangzhou).

### CD8^+^ T cell single-cell analysis

Raw data (fastq format) of single-cell transcriptomic were pre-analyzed by MobiVision V3.1 (MobiDrop), and reads were aligned to *Mus musculus* reference GRCm39. Filtered cell–gene matrix was obtained with MobiVision v3.2. In quality control, cells detected with more than 3 genes, less than 40,000 total counts, genes-by-counts ratio from 500 to 8,000, and mitochondrial genes ratio less than 20% were retained; genes detected in more than 3 cells were retained. Matrix was log-normalized and called 2,000 highly variable genes by omicverse Python package (v1.6.6) with Pearson’s method [56]. Dimensionality reduction and neighbor computation were performed by scanpy Python package (v1.10.0) with default parameters [57]. Unsupervised clustering was performed by Leiden algorithm with resolution 0.4. Cell subtypes were annotated based on their marker genes (Fig. 3O and P). Cytotoxic score was calculated based on the expression level of *Nkg7, Ccl4, Cst7, Prf1, Gzma, Gzmb*, and *Ifng* via geneset_aucell function of omicverse Python package [57,58]. Cytotoxic score was calculated based on the expression level of *Pdcd1, Ctla4, Tigit, Lag3, Havcr2*, and *Tox*. DEGs were calculated by rank_genes_groups function of scanpy Python package with default parameters [57]. DEG with false discovery rate (FDR) less than 0.01 and log_2_ fold change larger than 0.25 was used in gene ontology enrichment analysis, performed by GSEApy Python package DEG (v1.1.0) [59].

### Statistical analysis

All results are presented as the mean ± SEM. Statistical analysis was performed with Student’s *t* test (2-tailed unpaired) for comparison of 2 groups and the Kaplan–Meier method for assessing mouse survival. All the in vitro experiments were replicated at least 3 times. All the analysis was conducted using GraphPad Prism 9 unless specified otherwise. Differences were considered significant when *P* ≤ 0.05.

Full materials and methods are presented in Supplementary Materials and Methods. 

## Data Availability

All data supporting the findings of this study are provided within the paper and its supplementary materials. Sequence data included in this study have been deposited in the Gene Expression Omnibus of the National Center for Biotechnology Information, under accession number GSE283805 and GSE283806.

## Supplementary Materials

Supplementary 1 Materials and Methods Figs. S1 to S6 References
