# Supplementary material for: Protein Phosphatase 2A Promotes CD8+ T Cell Effector Function through the Augmentation of CD28 Costimulation
Source: Research (Wash D C). 2025 Jan 2;8:0545. doi: 10.34133/research.0545 (PMC11694323; doi:10.34133/research.0545)
Supplement: Supplementary 1 — Materials and Methods Figs. S1 to S6 References [file research.0545.f1.zip › 0929-SI Materials and Methods for research-research.0545 (1).docx]

**Supplementary Information for**

**Protein phosphatase 2A promotes CD8+ T cell effector function through the augment of CD28 co-stimulation.**

Kaixiang Zhu^1,2,3,*,#^, Deepak Rohila^4,5,*^, Yuanling Zhao^4,*^, Dmytro Shytikov^6^, Lize Wu^4^, Fan Zhao^4^, Shurong Hu^7^, Qin Xu^8^, Xuexiao Jin^4,#^, Linrong Lu^1,4,9,#^

^*^These authors contributed equally

^#^To whom correspondence should be addressed:

Linrong Lu: [lu_linrong@zju.edu.cn](mailto:lu_linrong@zju.edu.cn); Kaixiang Zhu: [zhu_kaixiang@zju.edu.cn](mailto:zhu_kaixiang@zju.edu.cn); Xuexiao Jin: shirleyj@zju.edu.cn.

866 Yuhang Tang Road, Medical Research Building A801, Hangzhou, 310058, P. R. China. Tel: 86-571-88981173;

**This PDF file includes:**

Material and methods

SI References

Legends for Figures S1-S6

**Material and methods**

**Mice and Cell lines**

*Ppp2ca* floxed mice were provided by X. Gao, Model Animal Research Center of Nanjing University. Mice with dLck-Cre and CD8a-Cre were purchased from The Jackson Laboratory. *Ppp2ca* knockout was confirmed by genotyping as described (*1*). Distal Lck-Cre and CD8a-Cre mouse genotype identification refers to Jackson's website (*2, 3*). Recombination-activating gene 1 (*Rag1*^-/-^) mice were purchased from GemPharmatech Co., Ltd. All mice were housed under specific pathogen-free conditions. Age and sex-matched mice were used in each experiment. All animal protocols were approved by the Review Committee from Zhejiang University School of Medicine. The B16F10 mouse melanoma cell line was provided by Professor Chenqi Xu, and the E.G7-OVA cell line was provided by Professor Dong Chen as a gift.

**Reagents**

Anti-CD4 (RM4-5), anti-CD4 (RM4-5 or GK1.5), anti-CD8a (53-6.7), anti-CD44 (IM7), anti-CD25 (PC61), anti-TCRβ (H57-597), anti-CD62L (MEL-14), anti-IFNγ (XMG1.2), anti-TNFα (MP6-XT22), anti-GZMB (GB11), anti-PD1 (J43), anti-CD69 (H1.2F3), anti-IL2 (JES6-5H4) and fixable viability dye (Invitrogen) et.al were used for flow cytometry;

Primary antibodies including anti-β-actin (Abclonal), anti-PP2A catalytic α subunit (BD bioscience), anti-PLCγ1 (Abcam), anti-Phospho-PLCγ1 (Bioworld), anti-p44/42 MAPK (ERK1/2) (Cell signaling), anti-Phospho-p44/42 MAPK (ERK1/2) (Thr202/Try204) (Cell signaling), anti-LCK (EMD Millipore), anti-Phospho-LCK (Cell signaling), anti-AKT (Cell signaling), anti-Phospho-AKT (Thr308) (Bioworld), anti-Phospho-AKT (Ser473) (Cell signaling) were used for immunoblotting. Supernatants from cell cultures were collected and the concentrations of IFNγ and TNF-α (all from Thermo Fisher) were determined according to the manufacturer’s instructions.

**Immunoblot of cell signaling**

Cells were lysed in cell lysis buffer (100 mM Tris-HCl, 4% SDS, 20% glycerol, 2% 2-mercaptoethanol, and 0.05% bromophenol blue, 1 mM PMSF, and protease inhibitors), the samples were then boiled and separated on 8-12% SDS-PAGE gels and transferred to nitrocellulose membranes (Millipore). The membranes were blocked with 5% BSA in TBST (0.5 M NaCl, Tris-HCl, pH 7.5, and 0.05% [vol/vol] Tween 20) for 1h at room temperature., ECL blotting reagents (Vazyme) were used for immunoblot detection after incubation with primary and secondary antibodies.

**Flow** **cytometry and cell sorting**

For cell surface marker staining, cells were stained with the relevant fluorescent antibody mixture at 4 ℃ for 30 min before analysis. For intracellular cytokine analysis, T cells were stimulated for 4 hours with phorbol 12-myristate 13-acetate (PMA, Beyotime) plus ionomycin (Beyotime) in the presence of Brefeldin A (Biolegend), cells were fixed with IC fixation buffer (eBioscience) after surface staining, then permeabilized with permeabilization buffer (eBioscience), stained with the relevant antibodies, and analyzed by flow cytometry. Samples were run on an LSR Fortessa (BD Biosciences) or NovoCyte (ACEA Biosciences). Cell sorting was performed by BD FACS Aria II cell sorter (BD Biosciences) or Beckman Moflo Astrios Eq. Data were analyzed using FlowJo (RRID: SCR_008520) v10.8.1 software (BD Biosciences).

***In vivo* animal model**

For acute virus infection model, WT and PP2A cKO mice were injected intraperitoneally with LCMV Armstrong (2 × 10^5^ PFU/mice). Mice were sacrificed 5 days after LCMV challenge, and splenocytes were harvested for the evaluation of immune response alterations and cytokine expression in both WT and PP2A cKO mice. For listeria monocytogenes infection, 5000 CFUs of listeria monocytogenes expressing the chicken ovalbumin (LM-OVA) were injected into mice and mice were sacrificed on day 7 after infection. Whole blood and splenocytes were isolated to measure antigen-specific CD8+ T cell response by flow cytometry.

For tumor engraftment, tumor cells were suspended in 100 μL PBS solution containing 50% Matrigel (Corning Inc). For engraftment to mice, 2 or 5 × 10^5^ tumor cells (B16F10 mouse melanoma cells) and 5 × 10^5^ tumor cells (E.G7-Ova mouse lymphoma cells) were subcutaneously injected into the right flank of mice (age 6-8 weeks). Tumor size was measured every two days using calipers and calculated as length × width, and mice were euthanized on day 15 for analysis as described below.

**Tumor infiltrating cell dissociation**

For isolation of tumor-infiltrating lymphocytes (TILs), the tumor was excised and digested in a 50mL tube for 20-30 min at 37° C with shaking at 120 rpm in HBSS containing calcium buffer with collagenase IV (1 mg/ml; sigma-tau) and DNase I (10 μg /ml; sigma-tau). Filter the suspension after digestion with a 70 μm cell strainer and crush any tissue clumps with a syringe plunger. Inactivate the enzyme activity by adding an equal amount of HBSS + 1% FBS and centrifuge at 350 × g for 10 minutes. Followed by performed the Percoll density gradient centrifugation. The mononuclear cells were removed from interphase, washed twice, and resuspended in HBSS supplemented with 2% FBS for further processing.

**Single-cell RNA-seq library preparation and sequencing**

CD8^+^ TILs were sorted by BD FACSAria Fusion sorter (BD Biosciences) and loaded into microfluidic chip of Chip A Single Cell Kit v2.1 (MobiDrop (Zhejiang) Co., Ltd., cat. no. S050100301) to generate droplets with MobiNova-100(MobiDrop (Zhejiang) Co., Ltd., cat. no. A1A40001). Each cell was involved into a droplet which contained a gel bead linked with up to millions oligos (cell unique barcode). After encapsulation, droplets suffer light cut by MobiNovaSP-100(MobiDrop (Zhejiang) Co., Ltd., cat. no. A2A40001) while oligos diffuse into reaction mix. The mRNAs were captured by cell barcodes with cDNA amplification in droplets. Following reverse transcription, cDNAs with barcodes were amplified, and a library was constructed using the High Throughput Single-Cell 3' Transcriptome Kit v2.1 (MobiDrop (Zhejiang) Co., Ltd., cat. no. S050200301) and the 3' Dual Index Kit (MobiDrop (Zhejiang) Co., Ltd., cat. no. S050300301). Libraries sequencing was performed on Illumina NovaSeq X Plus platform with 150 bp paired-end reads (Repugene Technology, Hangzhou).

***In vitro* stimulation and T-cell proliferation**

For stimulation, CD8+ naïve T cells purified from mice and then stimulated with plate-bound anti-CD3 antibody (eBioscience) and anti-CD28 antibody (Biolegend) for 1-3 days. Cells were stimulated for 4 hours with PMA plus ionomycin in the presence of Brefeldin A before cytokine analysis. The supernatants were collected for cytokine production analysis by ELISA.

For proliferation, CD8+ naïve T cells purified from mice were stained with CellTrace™ Violet CTV (Invitrogen) and then stimulated with plate-bound anti-CD3 antibody and anti-CD28 antibody for 3 days. The attenuation of CTV fluorescence was assessed using flow cytometry (FACS), and the supernatant was collected for cytokine production analysis.

**RNA sequencing and analysis**

Naïve CD8+ T cells from 6-week-old WT and PP2A Cα cKO mice were isolated and stimulated with anti-CD3 (0.1 ng/μL) plus anti-CD28 (3 ng/μL) for 1 h, cells were then lysed in TRIzol (Invitrogen). Total RNA was extracted and sequencing were processed by Novogene. Raw reads were processed through the genome alignment by STAR (RRID: SCR_004463) (v2.7.2a), and counted by featureCounts (RRID: SCR_012919) (v2.0.0)(*4, 5*). Differentially expressed genes (DEGs) were calculated by DESeq2 (RRID: SCR_000154) R-package (v1.40.1)(*6*). DEGs with *false discovery rate (FDR)* < 0.05, *fold change* > 1.5, and *baseMean*>1 were used in Gene Ontology (GO) term enrichment via clusterProfiler (RRID: SCR_016884) R-package (v4.8.1)(*7*). RNA sequencing results were plotted via gseapy Python-package (v1.1.1)(*8*).

**Statistical Analysis**

All results are presented as the mean ± SEM. Statistical analysis was carried out using Student’s t test (two-tailed unpaired) for two groups and the Kaplan-Meier method for mouse survival as indicated, all using GraphPad Prism 9 unless otherwise noted. Differences were considered significant when p ≤ 0.05.

**SI References**

1. Q. Xu *et al.*, Phosphatase PP2A is essential for T(H)17 differentiation. *Proc Natl Acad Sci U S A* **116**, 982-987 (2019).

2. Y. Maekawa *et al.*, Notch2 integrates signaling by the transcription factors RBP-J and CREB1 to promote T cell cytotoxicity. *Nat Immunol* **9**, 1140-1147 (2008).

3. Q. Wang, J. Strong, N. Killeen, Homeostatic competition among T cells revealed by conditional inactivation of the mouse Cd4 gene. *J Exp Med* **194**, 1721-1730 (2001).

4. A. Dobin *et al.*, STAR: ultrafast universal RNA-seq aligner. *Bioinformatics* **29**, 15-21 (2013).

5. Y. Liao, G. K. Smyth, W. Shi, featureCounts: an efficient general purpose program for assigning sequence reads to genomic features. *Bioinformatics* **30**, 923-930 (2014).

6. M. I. Love, W. Huber, S. Anders, Moderated estimation of fold change and dispersion for RNA-seq data with DESeq2. *Genome Biol* **15**, 550 (2014).

7. G. Yu, L. G. Wang, Y. Han, Q. Y. He, clusterProfiler: an R package for comparing biological themes among gene clusters. *OMICS* **16**, 284-287 (2012).

8. Z. Fang, X. Liu, G. Peltz, GSEApy: a comprehensive package for performing gene set enrichment analysis in Python. *Bioinformatics* **39**, (2023).

**Figure S1. The impact of PP2A deficiency on CD8^+^ T cell effector function.**

(A) The total cell number of effector CD8^+^ T cells (CD44^hi^CD62L^low^) in the spleen of WT or cKO mice (n = 3).

(B-D) Cell number count of GZMB^+^ CD44^+^ (B), IFNγ^+^ CD44^+^ (C), TNFα^+^ CD44^+^ (D) of CD8^+^ T cell in spleen (n = 3).

(E-F) Viral titer was measured in the spleen (E), and liver(F) on day 5 after infection (n = 3).

(G-J) Flow cytometry analysis of H2K^b^-OVA^+^ CD44^+^ CD8^+^ T cells in blood (G) and spleens (I) of control and PP2A-deficient mice 7 days after Listeria monocytogenes (LM-OVA) infection (n = 4). Flow cytometry analysis of IFNg/GZMB/TNFα producing CD8^+^ T cells (after ex vivo stimulation with OVA257-264 peptide) in blood (H) and spleens (J) of control and PPP2A-deficient mice 7 days after LM-OVA infection.

Mean ± SEM; **p* < 0.05, ***p* < 0.01, *** *p* < 0.001 two-tailed unpaired student’s t-test.

**Figure S2. Assessment of anti-tumor activity by PP2A deficient CD8^+^ T cells**

(A-B) The proportion of CD8^+^ TNFα^+^ (A) and CD8^+^ GZMB^+^ T cells (B) among TILs of WT or cKO mice challenged with E.G7 tumor cells. Representative plots (left) and statistics (right) are presented. (n = 6).

(C) CD8^+^ T cells were co-visualized with TUNEL by immunofluorescence staining of E.G7 tumor sections from WT and *Ppp2ca*^fl/fl^ */* dLck^cre^ mice. CD8 is shown in red, TUNEL in green. Scale bar 200 μm. (n = 6).

(D-E) Flow cytometry (left) was used to determine the expression (right) of PD-1 (D) and TIGIT in CD8^+^ T cells (E) among E.G7 TILs (n = 6).

(F) Flow cytometry (left) was used to detect the percentage (middle) and cell number count (right) of CD4^+^ T cells among TILs (n = 6).

(G) Schematic of the adoptive transfer assay *in vivo*.

(H-J) Representative plots of CD8^+^ T cells (H), CD8^+^ IFNγ^+^ T cells (I), CD8^+^ GZMB^+^ T cells (J) among E.G7 TILs.

(K) Flow cytometry (left) was used to determine the expression (right) of TIM-3 and TIGIT in CD8^+^ T cells (n = 6).
Mean ± SEM; **p* < 0.05, ***p* < 0.01, *** *p* < 0.001 two-tailed unpaired student’s t-test.

**Figure S3. Specific deletion of PP2A in CD8^+^ T cells does not change CD8^+^ T cells homeostasis.**

(A) Western Blot analysis of PP2A catalytic α subunit prepared from the lymphocyte populations sorted from thymus and lymph nodes.

(B-D) The percentage of CD4^+^ (B) and CD8^+^ T (C) cells in blood, LNs (lymph nodes), mLNs (Mesenteric lymph nodes) and spleen from WT and *Ppp2ca*^fl/fl^ */* CD8a^cre^ mice (6 weeks old, n = 5). Representative flow cytometry plots of T cell subsets from these organs are shown on (D).

(E-H) Quantification of naïve T cells (Tn, CD62L^hi^CD44^low^), Central memory T cells (Tcm, CD62L^hi^CD44^hi^) and effector memory T cells (Tem, CD62L^low^CD44^hi^) in Spleen (E), mLNs (F), and LNs (G). The representative plots of T cell subsets from these organs (H).

(I-J) WT and *Ppp2ca*^fl/fl^ */* CD8a^cre^ mice (8-14 weeks old) were subcutaneously injected with 5 × 10^5^ EG7 cancer cells subcutaneously and euthanized at 14 days post-implantation for analysis (n = 6). Representative plots of FACS (left) and the percentage (right) of CD8^+^ PD-1^+^ T cells in TILs (I). Flow cytometric (left) was used to assess the proportion (right) of CD8^+^ TIGIT^+^ T cells (J).

Mean ± SEM; ***p* < 0.01 two-tailed unpaired student’s t-test.

**Figure S4. Cell signaling in PP2A deficiency CD8^+^ T cells.**

(A) Naïve CD8^+^ WT and PP2A KO cells were stimulated with 0.1 ng/μL anti-CD3 plus 3 ng/μL anti-CD28 for 5 hours. Representative FACS (left) and the frequency (right) of CD69^+^ CD8^+^ T cells are shown.

(B) IL-2 levels were quantified using flow cytometry in naive CD8^+^ T cells isolated from WT and *Ppp2ca*^fl/fl^ */* CD8a^cre^ mice following a 2-day stimulation with anti-CD3 (0.1 ng/μL) + anti-CD28 (3 ng/μL).

(C) Naïve WT and PP2A deficient CD8^+^ T cells were isolated and stimulated with varying concentrations of CD3 and/or CD28 antibodies (0.3 ng/μL to 3.0 ng/μL, top), anti-CD3 alone (0.3 - 3.0 ng/μL, middle), or anti-CD3 (0.1 ng/μL) + anti-CD28 (0.3 - 3.0 ng/μL, bottom) for a duration of 72 hours. Flow cytometry was used to detect apoptosis.

(D) Naïve wildtype CD8^+^ T cells were isolated and stimulated with anti-CD3 (0.1 ng/μL) plus anti-CD28 (0.3 - 3 ng/μL) for 72 hours, and cells were treated with 2 μM of three PP2A inhibitor: Cantharidin (CAN, top), Okadaic Acid (OA, middle), and LB100 (bottom) in the meantime. Flow cytometry was employed for apoptosis detection.

(E-G) RNA-seq results of PP2A cKO mice. A. Volcano plot showing the differential expressed genes (DEG) of *in vitro* stimulated CD8^+^ T cells from *Ppp2ca* conditional knock out (cKO) and wild type (WT) mice (E). Dot size representing the mean expression level of each gene. Highlighted genes were marked with green lines. Heatmap showing the expression level of highlighted genes in each sample (F). Gene Ontology (GO) terms enriched by differential expressed genes showing the upregulated pathways in PP2A cKO and WT CD8^+^ T cells. Color bar representing the -Log10 (FDR) of each enriched term. Dot size representing the proportion of enriched genes to the gene set (G).

**Figure S5. PP2A promotes CD8 effector function by augmenting AKT phosphorylation.**

(A) Naïve CD8^+^ T cells were isolated from WT and *Ppp2ca*^fl/fl^ */* CD8a^cre^ mice and stimulated with anti-CD3 (0.5 ng/μL) for the indicated time points, and western blot was performed to detect total and phosphorylated AKT, ERK, PLCG1, LCK.

(B) Naïve PP2A-knockout CD8^+^ T cells and vehicle cells were stimulated with plate-bound anti-CD3 (0.1 ng/μL) and anti-CD28 (ranging from 0.03 to 1 ng/μL) in the presence of PBS or SC79 for specified durations. The expression profile of GZMB in these cells was analyzed using fluorescence-activated cell sorting (FACS) (top panel). Mean fluorescence intensity (MFI) of GZMB was quantitatively assessed (bottom panel).

(C-F) WT and PP2A-deficient naïve CD8^+^ T cells were stained with CTV and stimulated with anti-CD3 (0.1 ng/μL) plus anti-CD28 (in gradient 0.03-1 ng/μL) in the presence of PBS or SC79(C-D) / IN-1(E-F) for 72 hours. Expression profile of TNF-α and GZMB from the above-mentioned cells were analyzed by flow cytometry (left). MFI of TNF-α and GZMB was quantified (right).

Mean ± SEM; **p* < 0.05, ***p* < 0.01, *** *p* < 0.001 two-tailed unpaired student’s t-test.

**Figure S6. The expression and targeting of PP2A in tumor**

(A) The correlation of PP2A expression with the overall survival of kidney renal clear cell carcinoma, thymoma, ovarian serous cystadenocarcinoma and stomach adenocarcinoma. Data was obtained from KMplot.com.

(B, C) Representative plots of CD8^+^ T cell and IFNγ^+^ producing CD8^+^ T cells in E.G7 tumor (n = 4).
