# Supplementary figures and images for: Protein Phosphatase 2A Promotes CD8+ T Cell Effector Function through the Augmentation of CD28 Costimulation
Source: Research (Wash D C). 2025 Jan 2;8:0545. doi: 10.34133/research.0545 (PMC11694323; doi:10.34133/research.0545)

**Figure S1**

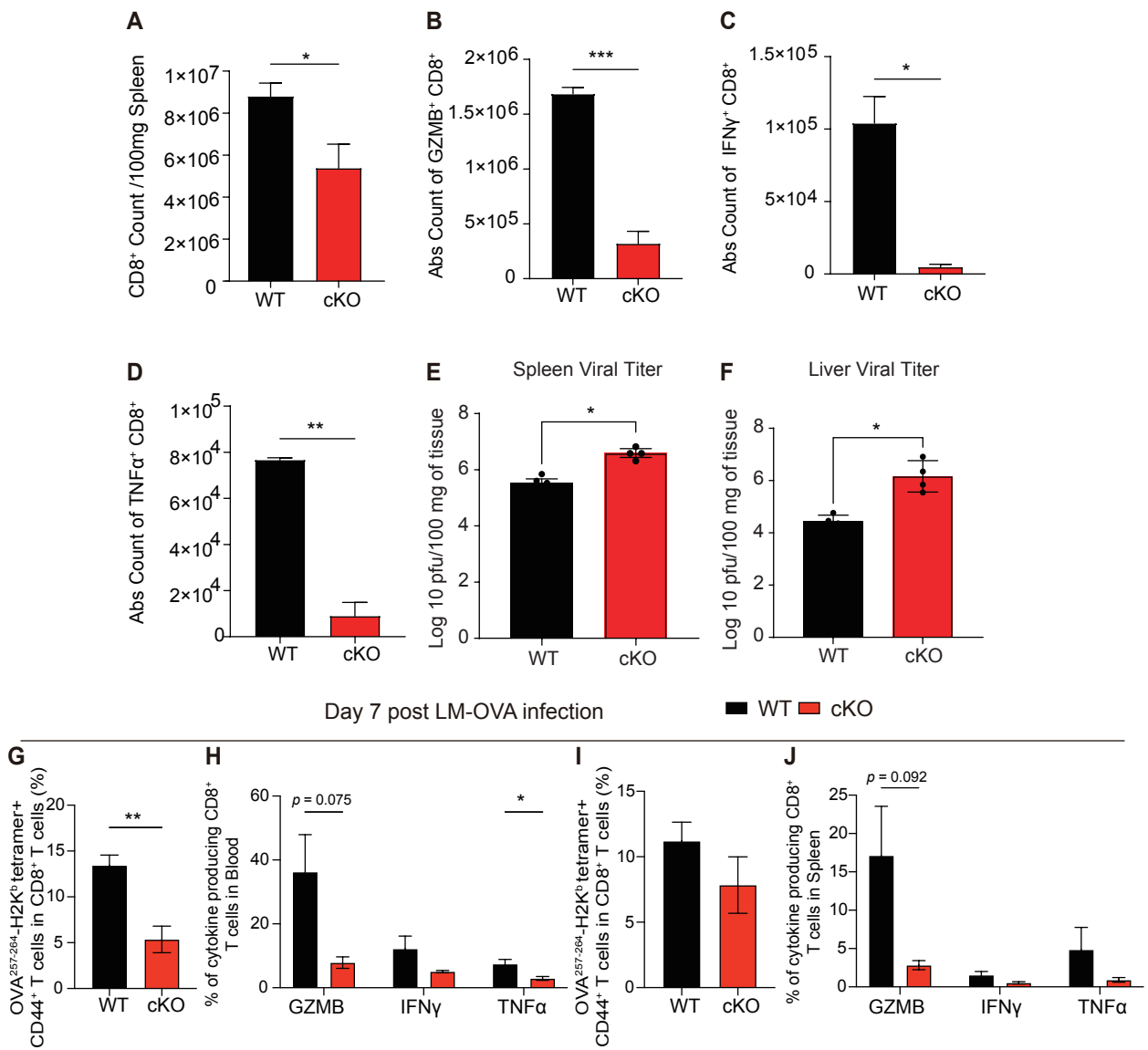

**Figure S2**

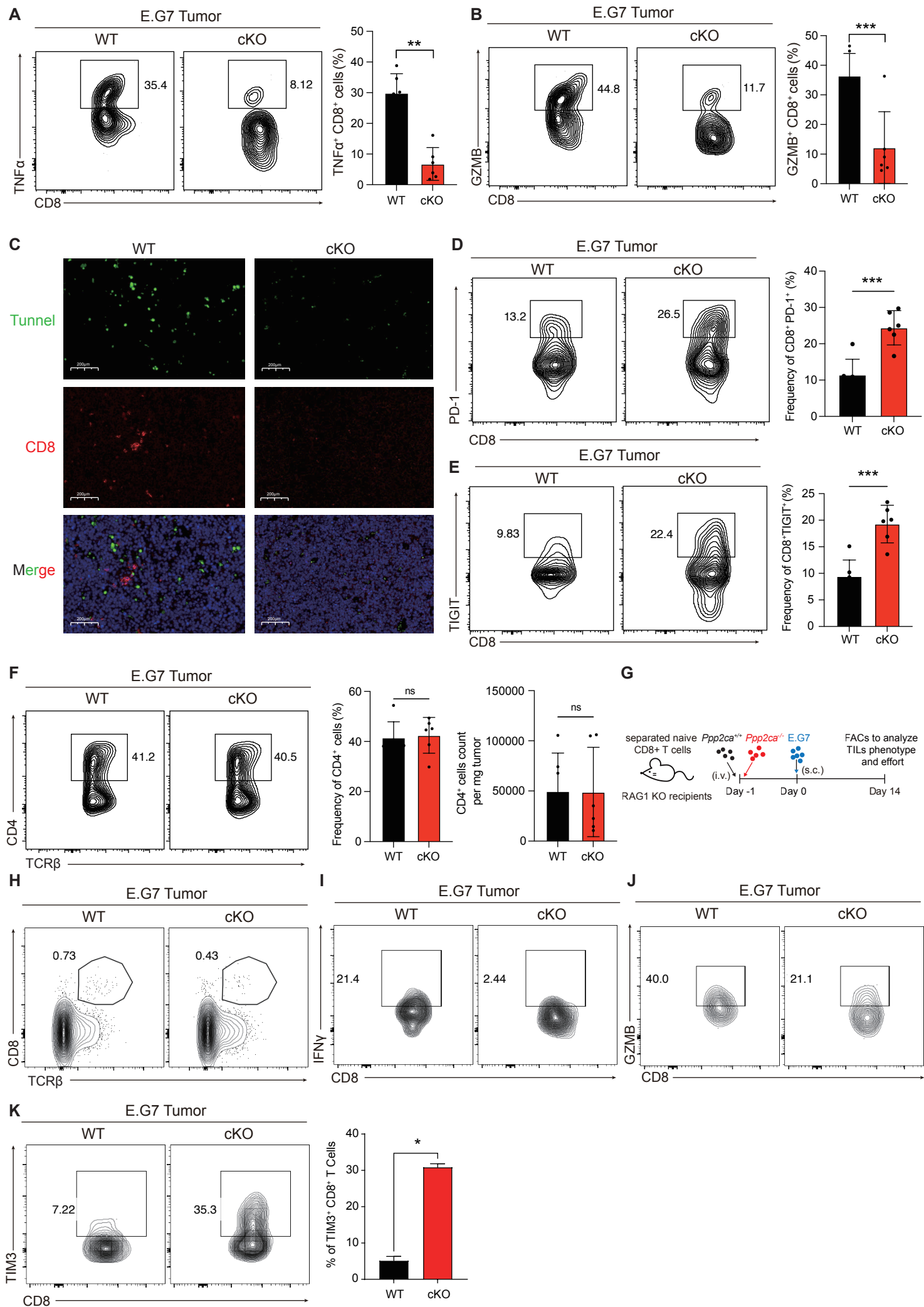

Figure S3

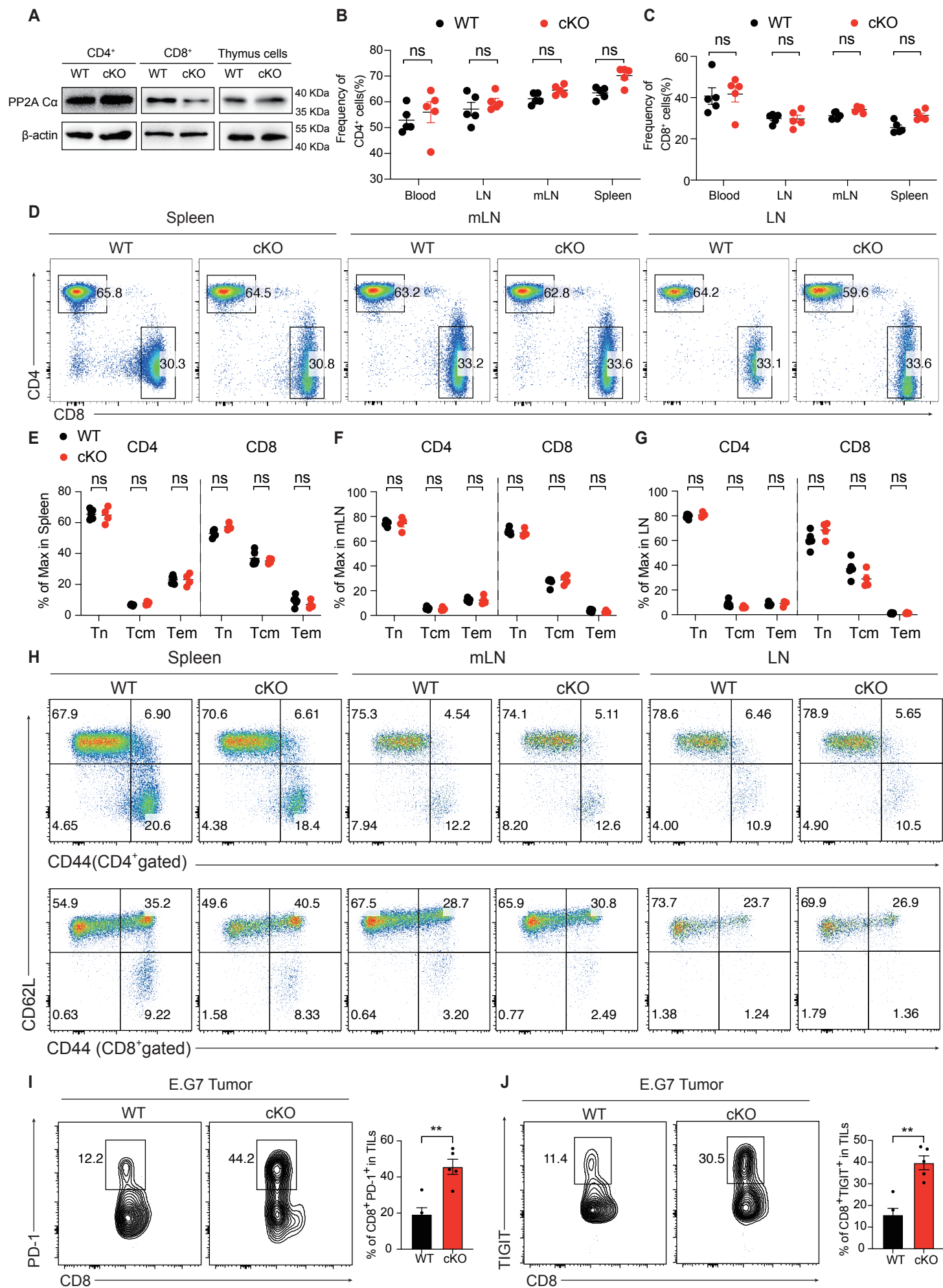

Figure S4

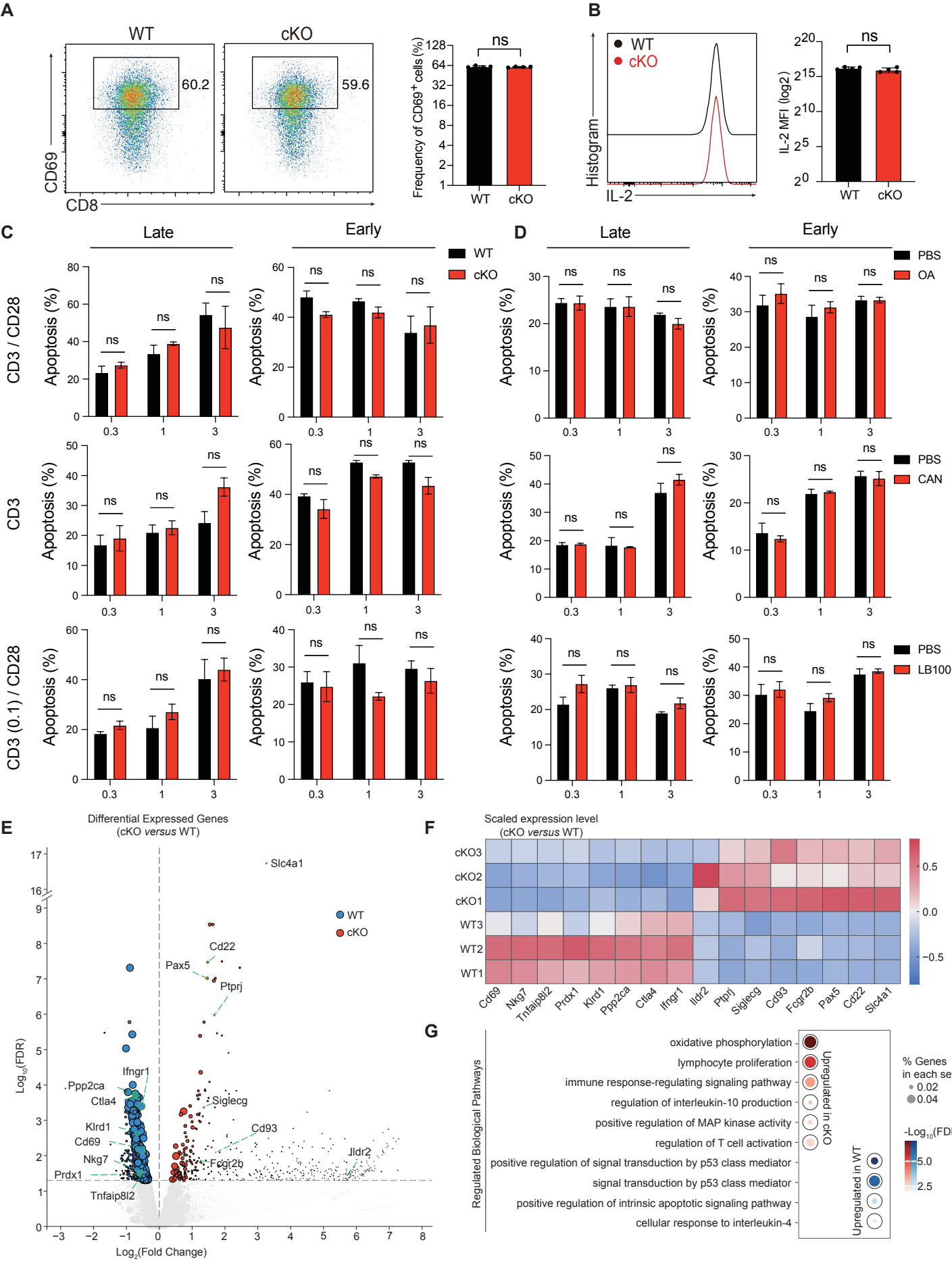

**Figure S5**

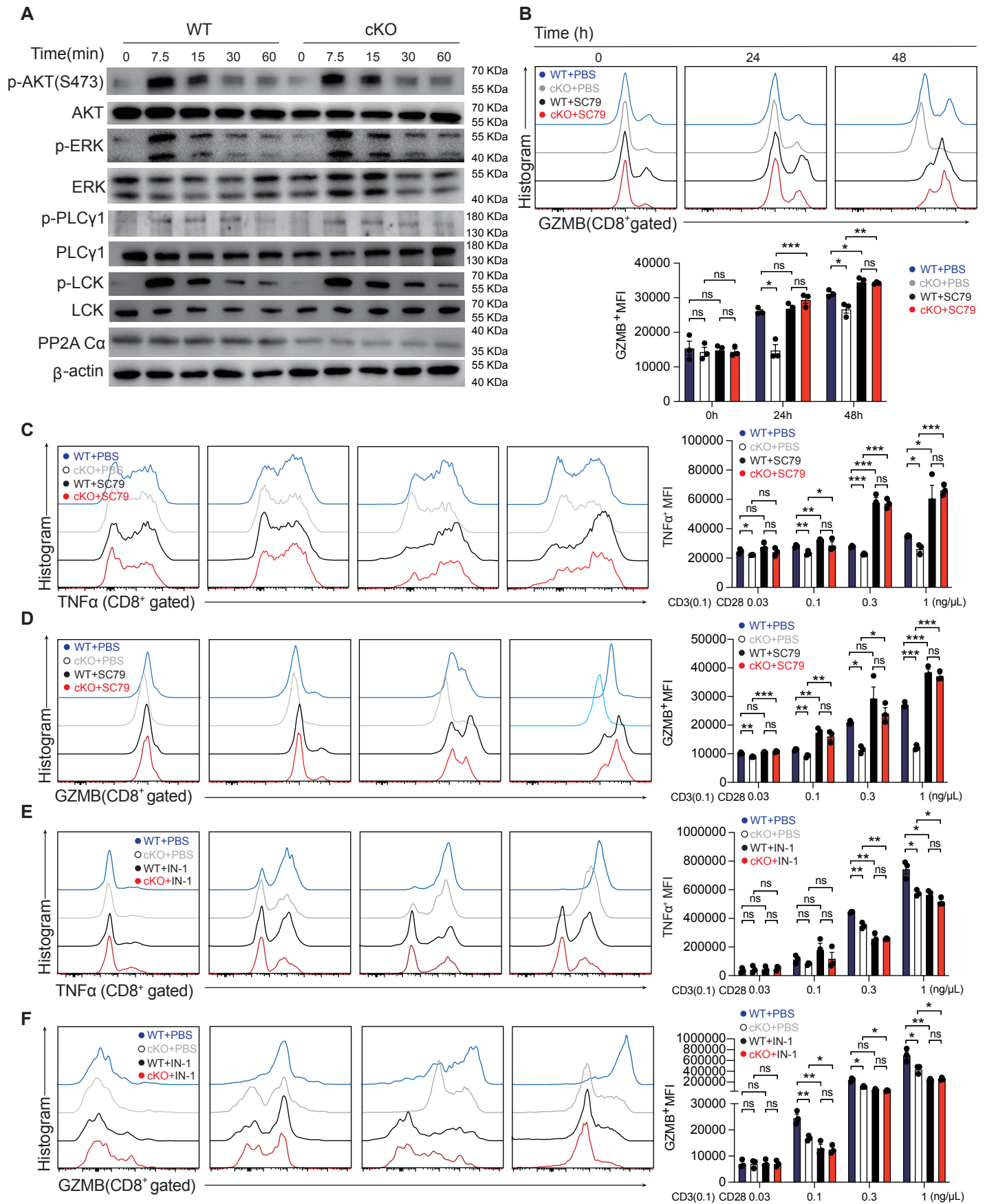

Figure S6

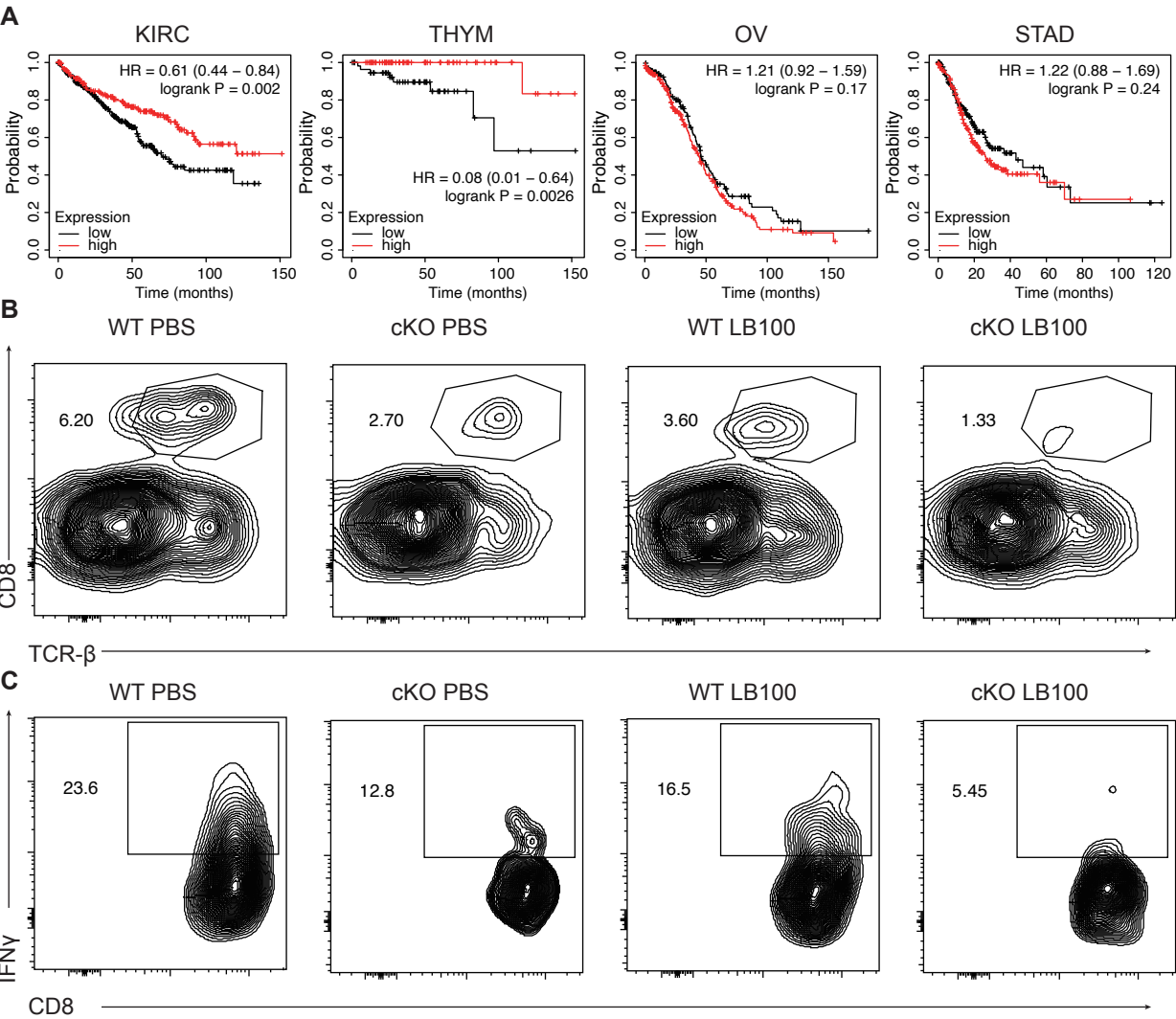

Supplement: Supplementary 1 — Materials and Methods Figs. S1 to S6 References [file research.0545.f1.zip › Figures S1-S6.pdf]
